# The first queen-worker association for Cretaceous Formicidae: the winged caste of *Haidomyrmex cerberus*

**DOI:** 10.3897/zookeys.1048.66920

**Published:** 2021-07-07

**Authors:** Yuanyuan Guo, Chungkun Shih, De Zhuo, Dong Ren, Yunyun Zhao, Taiping Gao

**Affiliations:** 1 College of Life Sciences and Academy for Multidisciplinary Studies, Capital Normal University, 105 Xisanhuanbeilu, Haidian District, Beijing 100048, China Capital Normal University Beijing China; 2 Department of Paleobiology, National Museum of Natural History, Smithsonian Institution, Washington, DC, 20013–7012, USA National Museum of Natural History Washington, DC United States of America; 3 Beijing Xiachong Amber Museum, 9 Shuanghe Middle Road, Beijing, 100023, China Beijing Xiachong Amber Museum Beijing China

**Keywords:** Alate queen, dealate queen, Haidomyrmecine, Myanmar, queen ant, workers

## Abstract

Two queen ant specimens, one alate and one dealate, from mid-Cretaceous (Late Albian–Early Cenomanian) Burmese amber are herein reported as belonging *Haidomyrmex cerberus* Dlussky, 1996. This is the first discovery and documentation of an alate queen in *Haidomyrmex*. Compared with workers of *Haidomyrmex cerberus*, alate and dealate queens are larger in body size, have smaller compound eyes, a longer antennal scape, more complex mandibles, and a relatively large-sized metasoma. It is hypothesized that these differences are due to caste differences.

## Introduction

Mandibles, as the main structures used for foraging, predation, food handling, defense and brood care (Hölldobler and Wilson 1990), are vital to the biology, taxonomy and evolutionary development of ants. For example, haidomyrmecine and zigrasimeciine ants with unique mandibles have been reported in mid-Cretaceous amber deposits from Myanmar (Dlussky 1996; Barden and Grimaldi 2012, 2013; Perrichot 2014; Perrichot et al. 2016, 2020; Miao and Wang 2019; Cao et al. 2020a, b, c; Lattke and Melo 2020). To date, ten genera and sixteen species of haidomyrmecine ants from Cretaceous amber deposits from France, Canada and Myanmar have been described. *Haidomyrmex*, as the type genus of the extinct subfamily Haidomyrmecinae, has been frequently discussed in relation to the other genera. In 1996, *Haidomyrmex cerberus* Dlussky, 1996, with a peculiar cranio-mandibular morphology, was found in Burmese amber and described. The combination of its bizarre mandibles and head capsule suggested that this ant might have been a specialized predator (Dlussky 1996). However, parts of the antennae, legs and gaster were not preserved in the type specimen whereas some key characters, especially those of the head, were obscured due to the turbidity of the amber piece. Cao et al. (2020a) provided a more detailed description of this species based on two additional worker specimens, including some characters of the antennae, head, legs and gaster. Based on some key characters, such as antennal length, size of compound eyes, location of trigger hairs, and mandible morphology, Lattke and Melo (2020) provided a key for identifying species of *Haidomyrmex*. Unfortunately, until now there was no description of alate queens in *Haidomyrmex*. Barden and Grimaldi (2012) described the only previously-known dealate queen, for the species *Haidomyrmex scimitarus* Barden & Grimaldi. In this study, we report two queen ant specimens of *Haidomyrmex cerberus*: one dealate and one alate, which represents the first discovery of a winged caste in *Haidomyrmex*. Comparing their morphological characters with those of the known dealate queen and workers provides insights into caste differences in *Haidomyrmex* ants.

## Material and methods

This study is based on two new amber specimens from the Hukawng Valley in the Kachin State of northern Myanmar, at the north end of Noije Bum at 26°15'N, 96°34'E, some 18 km south-west of the town of Tanai (Grimaldi et al. 2002; Cruickshank and Ko 2003). The deposit is dated to 98.79 ± 0.62 Mya based on radiometric uranium-lead dating (Shi et al. 2012). The recent finding of an ammonite embedded in amber and assignable to Puzosia (Bhimaites) supports a Late Albian–Early Cenomanian age of the amber (Yu et al. 2019). The newly-reported amber specimens are housed in the Key Lab of Insect Evolution and Environmental Changes, College of Life Sciences and Academy for Multidisciplinary Studies, Capital Normal University (**CNUB**), Beijing, China.

Specimens No. CNU-HYM-MA2015010 and No. CNU-HYM-MA2015011 are separately preserved in two yellow amber pieces with organic particles, tiny bubbles and dust covering the cuticle in places. Specimens were examined and photographed by using a Nikon SMZ 25 microscope equipped with a Nikon DS-Ri 2 digital camera. The line drawing and figure plates were prepared by using the Adobe Illustrator CC and Adobe Photoshop CC graphics software. Measurements were obtained using the measurement tool of the Nikon software. All measurements are provided in millimeters (mm). Measurements used in the descriptions, including their abbreviations, are detailed in Table 1.

**Table 1.** Measurements, including abbreviations, used in the descriptions.

Measurement	Explanation
Body length (BL)	in lateral view, from anteriormost point of head capsule excluding mandibles to posteriormost point of abdomen excluding sting.
Head length (HL)	in lateral view, from basal insertion of mandibles to the posteriormost point of head capsule.
Head height (Hh)	in lateral view, vertical distance from lowermost to highermost point of head capsule.
Scape length (SL)	maximum length of scape excluding condylar neck.
Eye length (EL)	maximum diameter of compound eye.
Mandible length (ML)	in lateral view, straight distance of mandible from basal insertion to apex.
Weber’s length (WL)	diagonal length of mesosoma in lateral view, from anteriormost point of pronotum to posteriormost point of propodeum.
Petiole height (PH)	maximum height of petiole excluding subpetiolar process in lateral view.
Petiole length (PL)	maximum length of petiole in lateral view.
Gaster length (GL)	maximum length of gaster (abdominal segments III–VII) in lateral view.

## Taxonomy

### Family Formicidae Latreille, 1809

**Subfamily Haidomyrmecinae Bolton, 2003**

#### Genus *Haidomyrmex* Dlussky, 1996

##### Haidomyrmex cerberus

Taxon classificationAnimaliaHymenopteraFormicidae

Dlussky, 1996

4BF19E05-20CA-5E33-A808-650EAE52AEF9

Figs 1 , 2 , 3

###### Specimens examined.

CNU-HYM-MA2015011, an alate queen, and CNU-HYM-MA2015010, a dealate queen, both housed in Capital Normal University, Beijing, (CNUB). ***Holotype*** NHM.In.20182, in Natural History Museum, London, UK.

###### Diagnosis.

Alate and dealate queens. Antenna with scape distinctly longer than pedicel and the two following flagellomeres combined, FII (second flagellomere) longer than each of the other flagellomeres. Labrum with two long setae curved upward. Mandibles long, internal surface of curved portion with a row of longitudinal serrations on the apical quarter; apical portion tapered to a blunt tip, external margin of apex each with a short erect and suberect seta. Maxillary palps distinctly elongate, formed of 6 segments; labial palps relatively short, formed of 4 segments. Propleuron well developed, with dorsal portion exposed and visible dorsally.

**Figure 1.**
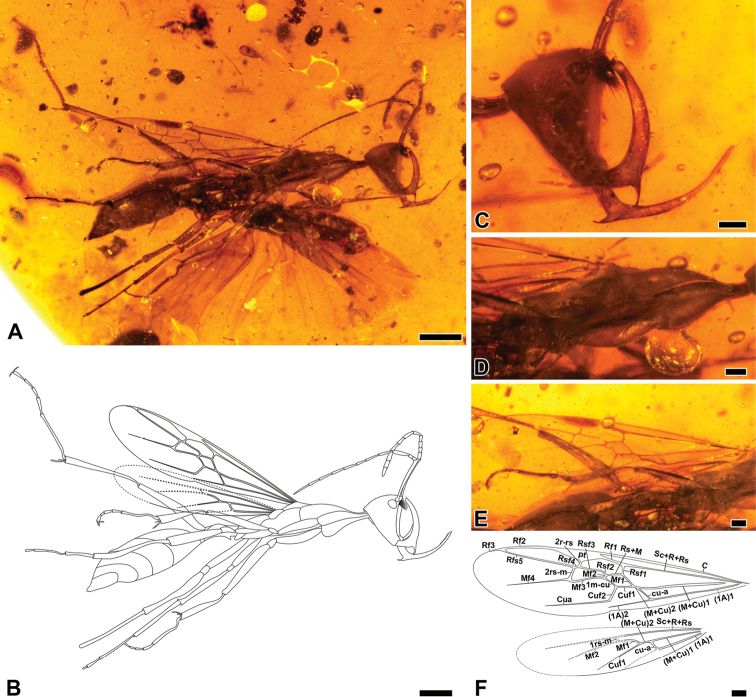
*Haidomyrmex cerberus*, alate queen specimen CNU-HYM-MA2015011 **A** photo of right lateral habitus **B** line drawing of habitus **C** photo of head in lateral view **D** photo of mesosoma in lateral view **E** photo of right forewing venation **F** line drawing of right forewing and right hind wing. Scale bars: 1 mm (**A, B**); 0.25 mm (**C–F**).

###### Description of alate and dealate queens.

Based on CNU-HYM-MA2015011, alate queen, with differential characters from CNU-HYM-MA2015010, dealate queen in square brackets.

***Head***: Vertex broad, evenly rounded, in lateral view approximately as high as long, shaped as an upside-down isosceles triangle; with sparsely thin erect setae [vertex in lateral view severely shrunken, glabrous]. No ocelli. Compound eyes situated high on head capsule, in lateral view ovoid and strongly convex [reniform and weakly convex]. Antennae inserted between compound eyes and flanking clypeal lobe, bases exposed and frontal lobes absent. Antenna geniculate, formed of 12 segments; scape ca. 8 times as long as pedicel [ca. 6 times], FI (first flagellomere) ca. 1.3 times as long as pedicel [ca. 1.2 times]; FII ca. 3 times as long as pedicel. Apex of scape slightly broadened, its margin bearing short and erect setae; FI with a long and curved seta on median ventral surface. Clypeal process a small lobe moderately protruding between bases of antennae, with short peg-like denticles above and longer, dense, stiff spine-like setae arranged in longitudinal rows on ventral half. Ventral surface of clypeus with one visible pair of long, fine trigger hairs [trigger hairs invisible]. Labrum with two long setae curved upward. Mandible long, scythe-shaped, internal surface of curved portion with 1–2 short setae near apex and a row of longitudinal serrations on apical quarter; apical portion tapered to a blunt tip, apex reaching clypeal lobe and each with one short, erect and suberect seta; ventral corner between basal and curved portion bearing a triangular blade, apparently symmetrical and with a single tooth; ventral margin of corner with sparsely fine setae from base to apex, becoming gradually shorter and thinner [with dense fine setae from base to one third of external surface of curved portion]. Maxillary palp exposed length 0.53, with 4 visible segments [length 0.86, with 6 obvious segments]. Labial palp is invisible [length 0.30, with 4 segments].

**Figure 2.**
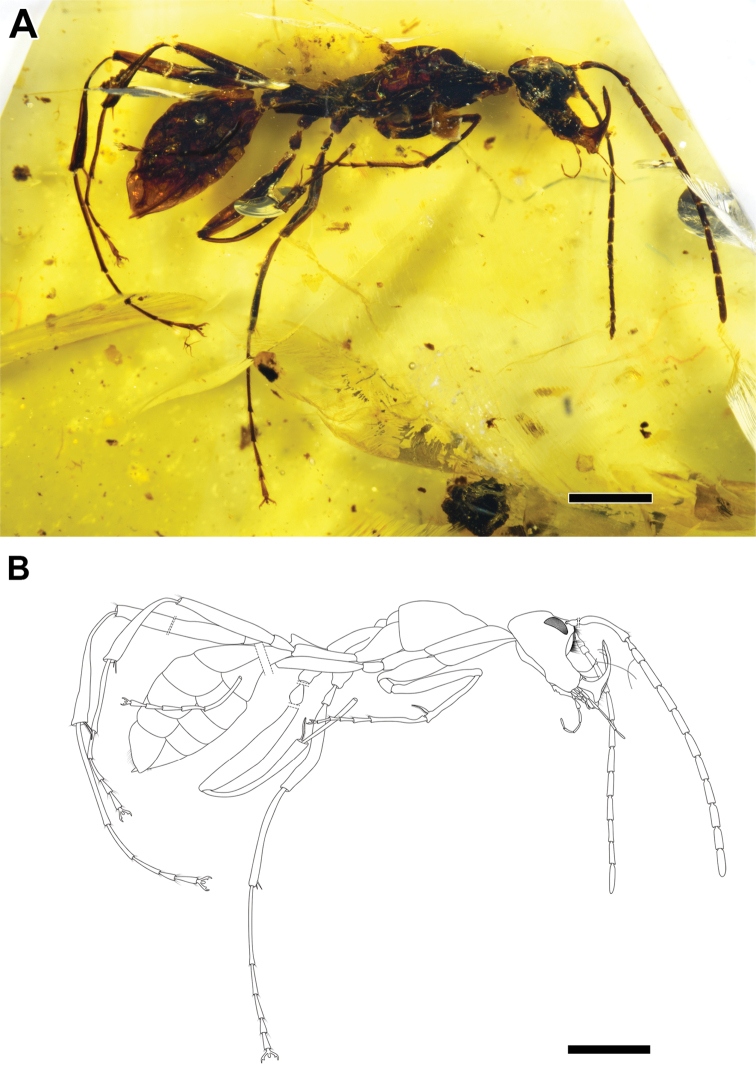
*Haidomyrmex cerberus*, dealate queen specimen CNU-HYM-MA2015010 **A** photo of right lateral habitus **B** line drawing of habitus. Scale bars: 1 mm.

***Mesosoma***: Long, slender, with sparsely thin erect setae [no visible setae]. Neck narrow and short, pronounced in lateral view. Propleuron well developed and visible in lateral view. Pronotum well developed, convex in anterior two thirds, gradually flattened in posterior third, extending laterally to anterior level of procoxa. Sulcus between pronotum and propleuron and between pronotum and mesonotum present, complete. Mesoscutum shorter than pronotum, mesoscutal dorsal outline slightly convex, with parapsidal furrows converging posteriorly to reach anterior mesonotal margin. Mesoscutellum posteriorly expanded, dorsal and posterior mesoscutellar surfaces concave. Dorsal level of metanotum and propodeum nearly at same level; propodeum slightly lower in elevation and dorsal surface gradually sloping posteriorly [metanotum and propodeum gradually sloping posteriorly]. Metapleural gland opening oval-shaped, slightly depressed. Legs long. Length of procoxa: 1.24 [0.70]; mesocoxa: 1.03 [0.38]; metacoxa:1.32 [0.47]; protrochanter: 0.20 [0.17]; meso- and metatrochanters: 0.27 [0.20]; profemur: 1.65 [1.17]; meso- and metafemora: ca. 1.78 [1.40]; protibia: 1.23 [0.94]; mesotibia: 1.32 [1.25]; metatibia: 1.92 [1.47]. Protibia apex with one large pectinate spur and two short spine-like setae, pecten of probasitarsus with fine hairs of uniform length. Mesotibia with two simple spurs, metatibia with one large pectinate spur and one short simple spur. Protarsus length 1.80 [1.40]; mesotarsus incompletely preserved, part of tarsomeres absent [complete, length 2.09]; metatarsus length 2.65 [2.55]. Ventral surface of tarsomeres with fine setulae and apex of tarsomeres I–V with two pairs of fine, long setae. Pretarsal claws with distinct subapical teeth; arolium small.

***Metasoma***: Petiole ca. 2.5 times as long as height [ca. 2.2 times], petiolar tergite a broadly convex node, with anterior surface approximately twice as long as posterior surface, with short anterior peduncle; small subpetiolar process projecting ventrally as a small triangle. Gaster with five segments, gastral segments I and II (abdominal segments III and IV) ca. 0.50 of total gaster length. Pygidium and hypopygium setulose. Sting very well developed.

***Right forewing***: Venation almost complete, anterior margin slightly folded. Cell 1R1C/SMC1 hexagonal; cell 1MC/DC1 with five sides, Rsf2 and Rsf3 distinguished, Rsf4 very short, almost as long as Mf2; (M+Cu)1 branched into (M+Cu)2 and cu-a; (M+Cu)2 short, nearly half of Rsf1; Rs+M almost as long as Mf1 and almost parallel to Cuf1; Mf2 present to juncture of Rs+M and 1m-cu; cross-vein 2rs-m slightly oblique. Nearly whole right hind wing folded over itself. (M+Cu)2 nearly as long as cross-vein cu-a; Mf1 aligned with Mf2 [wings not preserved].

###### Measurements

**(in mm).** (CNU-HYM-MA2015011, alate queen), [CNU-HYM-MA2015010, dealate queen]. BL (7.75) [6.31]; HL (1.15) [1.17]; Hh (1.24) [0.96]; EL (0.24) [0.28]; length of antennomeres (total 4.41, scape 1.12, pedicel 0.13, FI 0.18, FII 0.39, FIII 0.34, FIV 0.33, FV 0.31, FVI 0.32, FVII 0.34, FVIII 0.33, FIX 0.29, FX 0.30) [total 3.91, scape 0.75, pedicel 0.12, FI 0.15, FII 0.39, FIII 0.35, FIV 0.33, FV 0.28, FVI 0.31, FVII 0.31, FVIII 0.30, FIX 0.28, FX 0.33]; ML (0.98) [0.64]; WL (3.01) [2.45]; PL (0.79) [0.51]; PH (0.31) [0.23]; GL (2.78) [2.24].

###### Remarks.

Assignment of these two new specimens to *H. cerberus* is based on most of the characters used by Barden and Grimaldi (2012) and Cao et al. (2020a). This species is most similar to *H. scimitarus*, but the two new specimens could be assigned to *H. cerberus* by having 1) a slightly longer scape, longer than the pedicel and the two following flagellomeres combined (vs. scape visibly shorter in *H. scimitarus*); 2) labrum with two long setae curved upward (vs. labrum with four fine setae); 3) ventral corner of mandible between basal and curved portion with a triangular blade, apparently symmetrical and with a single tooth (vs. 3–4 fine mesal teeth on left mandible, 2–3 slightly larger teeth on right mandible); and 4) head with sparse thin and erect setae (vs. glabrous in *H. scimitarus*).

**Figure 3.**
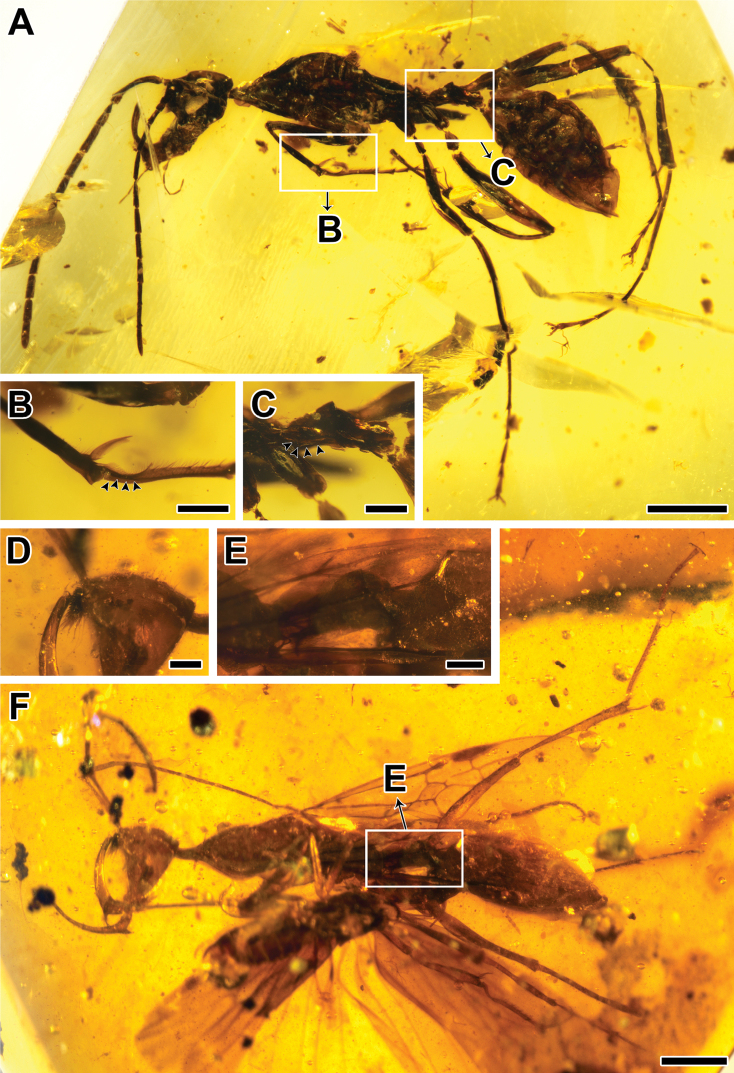
*Haidomyrmex cerberus*, specimens CNU-HYM-MA2015010 (**A–C**) and CNU-HYM-MA2015011 (**D–F**) **A** photo of left lateral habitus **B** photo of protibial apex and associated tarsus **C** photo of petiole in lateral view **D** photo of clypeal lobe in lateral view **E** photo of petiole in lateral view **F** photo of left lateral habitus. Scale bars: 1 mm (**A, F**); 0.25 mm (**B–E**).

## Conclusion

Most workers and queens of modern ants are known and the castes can be differentiated by body size and by minor aspects of the mandibular morphology (Hölldobler and Wilson 1990). It is highly probable that all the differences between the two queens described herein and the workers of *H. cerberus* revised by Cao et al. (2020a) are simply due to the difference in caste. The two queens can be differentiated from workers of *H. cerberus* by 1) the larger body of 6.3–7.8 mm (vs. 4.5–5.0 mm body lengths for workers); 2) the obviously longer scape, distinctly longer than the pedicel and two following flagellomeres combined (vs. scape as long as the pedicel and two following flagellomeres combined) and ca. 6–9 times as long as pedicel (vs. ca. 4 times as long as pedicel); 3) the more complex shape of the mandibles, with inner surface with a row of longitudinal serrations near the apex, and triangular blade clearly longer and sharper; 4) metasoma relatively large in proportion to total body size (ca. 0.36 times as long as body vs. ca. 0.32 times as long as body), because of flight muscles. Queens of modern species usually have larger eyes relative to head size compared to workers. Surprisingly, the queens of *H. cerberus* have smaller compound eyes (diameters of 0.24 mm and 0.28 mm) than those of workers (0.30 mm). Documentation of these differences contributes to a better understanding of the Cretaceous Formicidae and shows differences among castes of *Haidomyrmex cerberus* for the first time.

## Supplementary Material

XML Treatment for Haidomyrmex cerberus
